# Leukocytoclastic Vasculitis Occurring During Upadacitinib Therapy in Ulcerative Colitis: A Case Report

**DOI:** 10.5152/tjg.2026.25681

**Published:** 2026-01-28

**Authors:** Yunus Günegül, Mukaddes Tozlu, Yunus Emre Demiral, Ahmet Tarık Eminler, Mustafa İhsan Uslan

**Affiliations:** Department of Gastroenterology, Sakarya University Faculty of Medicine, Sakarya, Türkiye

To the Editor,

Upadacitinib, a selective Janus kinase 1 (JAK1) inhibitor, is an effective treatment for moderate-to-severe ulcerative colitis.^1^ Although several adverse reactions have been reported with upadacitinib, leukocytoclastic vasculitis (LCV) has not been specifically described in patients with ulcerative colitis (UC) receiving this therapy. Herein, we report a biopsy-confirmed case of LCV that developed during upadacitinib therapy in a patient with UC.

A 54-year-old man with a known case of UC with a 7-year history was admitted for flares. At the time of diagnosis in 2017, the disease extent was E2S2 (moderate, left sided). Informed consent was obtained. The patient initially achieved remission with corticosteroids and mesalazine followed by maintenance therapy with mesalazine. In October 2022, he was started on azathioprine at a dosage of 2.5 mg/kg/day p.o. and adalimumab at 40 mg subcutaneously every 2 weeks after the second flare-up. After 12 weeks of treatment without a therapeutic response, the patient was identified as a primary nonresponder to adalimumab. Consequently, both adalimumab and azathioprine therapy were discontinued. In January 2023, he started induction therapy with vedolizumab (300 mg at weeks 0, 2, and 6, followed every 8 weeks). The patient remained in clinical remission after treatment with vedolizumab for over one year. However, in December 2024, the patient was readmitted because of disease exacerbation. Upadacitinib was initiated at an induction dose of 45 mg/day p.o. for 8 weeks, followed by a maintenance dose of 15 mg/day. Approximately 4 months later, the patient presented with non-blanching purpuric and petechial lesions on both lower extremities and the dorsum of his feet (Figure 1). Two days later, the rash progressed and spread to involve the trunk and upper extremities. At the time of presentation, there were no associated systemic symptoms, such as fever, joint pain, weight loss, or gastrointestinal symptoms.

Colonoscopy revealed mildly erythematous mucosa and loss of vascular pattern, with 3-4 pseudopolyps in the rectum and sigmoid colon, corresponding to a Mayo endoscopic subscore of 1 (Figure 2). Laboratory analysis results were consistent with those of biochemical remission.

A comprehensive evaluation of autoimmune, infectious, hepatic, and renal function was conducted. Tests for antinuclear antibodies, antineutrophil cytoplasmic antibodies (ANCA), rheumatoid factor, anti-cyclic citrullinated peptide antibody, and serologies for Epstein–Barr virus, cytomegalovirus, and herpes simplex virus types 1 and 2 were negative. Additionally, serological tests for hepatitis A, B, C, and HIV were negative. The levels of complement proteins C3 and C4 were normal. The liver enzyme levels and renal function were normal. Thoracic and abdominal computed tomography revealed no signs of systemic vasculitis, malignancy, or lymphoproliferative disease.

A punch biopsy of a lesion in the lower extremity showed subepidermal edema, dense perivascular neutrophilic infiltration, leukocytoclasia, and fibrinoid necrosis of the vessel walls. These results were consistent with LCV. Notably, no evidence of granulomatous inflammation or vascular thrombosis was found.

Upadacitinib was promptly discontinued and intravenous methylprednisolone was initiated at a dose of 1 mg/kg/day. A rapid and significant improvement in the skin lesions was observed. The patient was then switched to ustekinumab for long-term maintenance therapy. At the follow-up appointment, the skin lesions resolved and the patient was in clinical and biochemical remission. The patient was not taking any other medications and had no new exposures or comorbidities that could account for the vasculitic eruption.

Compared to conventional treatments such as aminosalicylates, corticosteroids, and immunomodulators, upadacitinib is a newer therapeutic option for UC.^2^^,^^3^ Its rapid onset, oral administration, steroid-sparing effect, and ability to induce remission in severe cases have made it a preferred treatment for UC. Although its long-term efficacy and safety are well established in rheumatologic conditions, such as rheumatoid arthritis and psoriatic arthritis, findings may vary across diseases and populations.^4^ Despite its favorable profile, upadacitinib has been associated with adverse events, including serious infections, thromboembolic complications, and dermatological reactions. The cutaneous side effects include acneiform eruptions, maculopapular rashes, and herpes zoster. However, vasculitic skin reactions, such as LCV, have not been reported in trials or post-marketing data.^4^^,^^5^

Leukocytoclastic vasculitis is a small-vessel vasculitis characterized by neutrophilic infiltration and fibrinoid necrosis of vessel walls. Alternative causes of LCV, including infections, systemic autoimmune diseases, malignancies, and systemic vasculitides, should be systematically evaluated; in the present case, these were reasonably excluded based on the clinical, laboratory, imaging, and histopathologic findings.^6^^,^^7^ Drug exposure is a well-recognized contributor to LCV, and recently initiated therapies should be carefully reviewed when evaluating potential triggers. Delayed-onset vasculitic reactions have been described in association with biologic therapies; however, latency periods may vary across different therapeutic classes and mechanisms.^8^

Leukocytoclastic vasculitis is a rare extraintestinal manifestation (EIM) of UC, typically observed during active disease and often associated with ANCA positivity and other EIMs.^9^ In contrast, our patient had negative ANCA test results, no additional EIMs, and clinical and biochemical remission with no evidence of significant active disease at the time of onset, making a UC-related vasculitic process less clearly supported by the available clinical findings. Nevertheless, even low-grade mucosal inflammation may contribute to a permissive immunologic milieu; however, in the absence of systemic inflammatory activity, it may be insufficient on its own to account for the development of LCV. In this clinical context, alternative contributing factors, including medication-related effects, warrant careful consideration.

In conclusion, we report a biopsy-confirmed case of LCV occurring during upadacitinib therapy in a patient with UC. While causality cannot be definitively established, this case highlights the importance of clinical awareness regarding rare cutaneous vasculitic reactions during JAK inhibitor therapy.

## Figures and Tables

**Figure 1.**
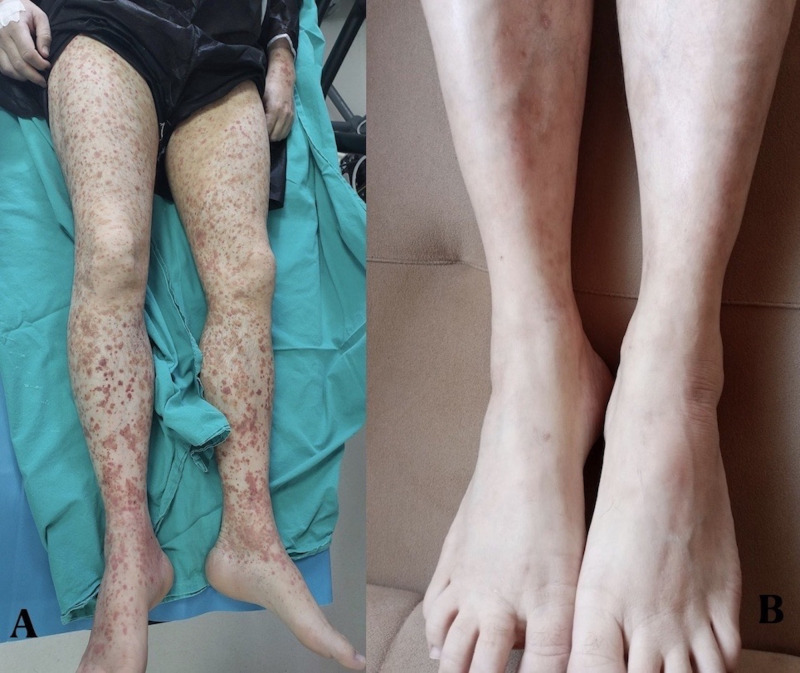
(A) Non-blanching purpuric and petechial lesions predominantly involving both lower extremities and dorsum of the feet. (B) Follow-up image demonstrating complete resolution of the cutaneous lesions.

**Figure 2.**
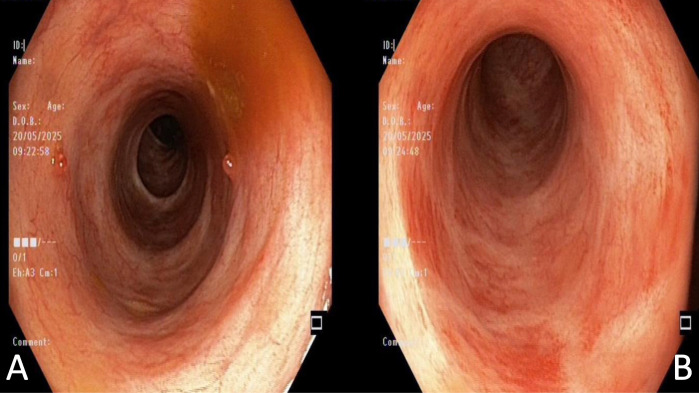
(A) Colonoscopic image showing pseudopolyps and mucosal edema in the rectosigmoid region. (B) Colonoscopic image showing erythematous mucosa with mild friability.

## Data Availability

The data that support the findings of this study are available on request from the corresponding author.
